# Hospital Saturday Fund

**Published:** 1905-04-08

**Authors:** 


### HOSPITAL SATURDAY FUND.

### ANNUAL GENERAL MEETING.

The annual general meeting of the Hospital Saturday Fund took place on Saturday, 1st inst., at the offices, 54 Gray's Inn Road, Mr. W. V. Stratford, Chairman of the Collection Committee, being voted to the chair. The report and accounts for 1904 were presented. The report stated that the collection was again a record one, the income being the highest in the history of the Fund—*i.e.*, £24,344, an increase of £670 on that for the previous year. The amount received from the local committees was £2,589, an increase of £363, and St. Marylebone, Camberwell, Clapham and Balham, Southwark, Poplar, Walthamstow, Enfield, Greenwich, and Deptford received special commendation, the first-named sending in £317, and the last £109. The standing committees had continued to perform their important duties satisfactorily, and 92 full committee and 113 sub-committee meetings were held during the year. There was still a debt of £1,499 1s. 6d. on the Premises Fund, £534 16s. 1d. having been paid off during the year. The distribution committee issued 36,614 letters, including 1,609 for convalescent homes, of which 660 were on the part-payment system. Towards the cost of the 4,644 appliances supplied, the patients contributed £2,289 9s. 4d. The supply of artificial teeth was still restricted to subscribers or their wives, and applications were so numerous that many were required to pay the full cost, while in no case was less than three-quarters charged. One hundred and fifty ambulance boxes were in use, and 5,474 first-aid cases were reported, 319 of which were removed to hospitals. Notwithstanding increased official work, the management expenses did not exceed 9·65 per cent. The committee were glad to note that the work of the National Committee for the Establishment of Sanatoria for Workers Suffering from Tuberculosis, which was inaugurated by the Fund, was making satisfactory progress. The committee had decided to purchase a site of 250 acres at Benenden in Kent, and it was hoped that in the near future a sanatorium to accommodate 200 patients would be established. The board unanimously decided to contribute £500 towards the building fund, and to secure 15 beds for the use of its supporters. Mr. A. J. Dear, in moving the adoption of the report and accounts, said the thanks of the supporters were specially due to the auditors, Messrs. W. H. Pannell and Co., whose gratuitous labours represented a donation of at least £100 annually. The motion was carried unanimously. The Lord Mayor having been elected president for 1905, and the hon. officers re-elected, the proceedings terminated with the usual votes of thanks.

At the quarterly meeting which followed, the reports of the various standing committees were received. Considerable discussion arose on that presented by the distribution committee, which stated that a deputation appointed to visit the Royal Eye Hospital to make enquiries into the arrangements for out-patients were convinced that everything was being done for their accommodation under present circumstances, and that a cheque for the award had been handed to the secretary. Several speakers complained that the arrangements were not satisfactory, early applicants having to wait in a narrow passage, where they were in danger of being run over, and contending that it was merely a question of opening a gate. The deputation said they believed better arrangements were under consideration by the hospital authorities, and in the meantime the committee considered it would be an injustice to withhold the grant. On a vote being taken, the report was adopted, with four dissentients. Recommendations to grant diplomas of merit to William Blanks and William Robert Street for conspicuous first-aid services under specially trying circumstances were confirmed; the first recipient was a shunter on the Midland Railway, and the second was recovering from typhoid fever at the time of rescuing a child from the water. Reports on visits paid by delegates to the Morley House and Alfred Bevan Convalescent Homes having been adopted, it was announced that the annual public meeting of the Fund would take place at the Mansion House on Saturday, the 29th inst., at 3.30, when Dr. Latham would deliver an address on "Sanatoria for Workers."

